# Ontogeny shapes the ability of ETV6::RUNX1 to enhance hematopoietic stem cell self-renewal and disrupt early lymphopoiesis

**DOI:** 10.1038/s41375-024-02149-2

**Published:** 2024-01-19

**Authors:** Mohamed Eldeeb, Anna Konturek-Ciesla, Qinyu Zhang, Shabnam Kharazi, Johanna Tingvall-Gustafsson, Jonas Ungerbäck, Mikael Sigvardsson, David Bryder

**Affiliations:** 1 Division of Molecular Hematology, Department of Laboratory Medicine, Lund Stem Cell Center, Faculty of Medicine, Lund University, 221 84 Lund, Sweden; 2 BKV, Linköping University, 581 83 Linköping, Sweden

**Keywords:** Haematopoiesis, Haematopoietic stem cells, Acute lymphocytic leukaemia, Haematopoietic stem cells

## To the Editor:

The ETV6::RUNX1 (E/R) translocation is the predominant chromosomal aberration in pediatric acute lymphoblastic leukemia (ALL) [1]. Despite its good prognosis, current treatments impose long-term side effects and a 20% relapse rate [1, 2], emphasizing the importance of understanding disease mechanisms for improved treatments.

E/R leukemogenesis begins in utero upon acquisition of the fusion gene. Despite the high prevalence of this initial event, only a minority acquire secondary mutations and progress to overt disease, with infections possibly playing a role as triggers [1]. The period between the initial and secondary events can extend over a decade, emphasizing a remarkable longevity of the preleukemic cells [1]. The specific cell of origin for E/R leukemia is debated but likely arises from an undifferentiated hematopoietic stem/progenitor cell (HSPC) [1, 3–5].

Here, to gain new insights into the E/R preleukemic state, we generated a transgenic inducible mouse model (iE/R) that enables reversible induction of E/R (Supplementary Fig. S1A). To confirm the model’s inducibility and expression levels, we performed quantitative reverse-transcription PCR (qRT-PCR) on RNA extracted from either cultured (Supplementary Fig. S1B) or fresh bone marrow (BM) cells (Supplementary Fig. S1C). This verified E/R expression only upon Dox administration, at levels closely matching those of REH cells, a human cell line for E/R leukemia (Supplementary Fig. S1C).

Most E/R-ALL patients present with inactivation of genes critical for normal B-cell development, such as Pax5 and Ebf1 [6]. To assess the impact of E/R on B-ALL, we introduced the M2 reverse Tetracycline transactivator (M2-rtTA) and iE/R alleles into Pax5^+/−^Ebf1^+/−^ mice [7]. Unfractionated E/R Pax5^+/−^Ebf1^+/−^ BM cells were then transplanted into recipient mice, both with and without Dox treatment. This revealed that E/R expression significantly accelerated B-ALL development (Supplementary Fig. S1D).

By transplanting unfractionated wild-type (WT) BM cells into lethally irradiated iE/R mice, we next examined the influence of E/R expressing non-hematological cells on hematopoiesis. This revealed no significant alterations in hematopoietic BM compartments, including on B-cell frequencies (Supplementary Fig. S1E–H), arguing that E/R alters hematopoiesis by mechanisms intrinsic to hematopoiesis.

**Fig. 1:**
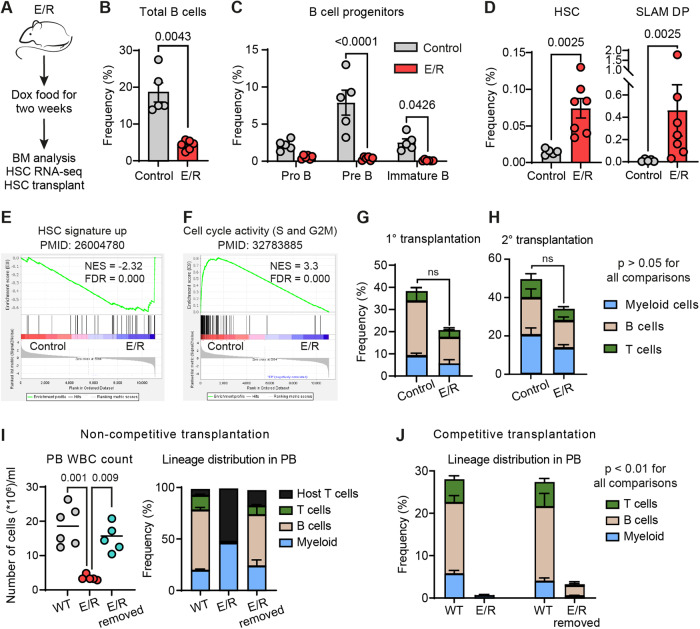
E/R-induced HSCs persist in the bone marrow with impaired hematopoietic reconstitution and lymphoid differentiation potentials that are restored upon cessation of E/R expression. **A** Experimental setup for **B**–**G** panels. **B** Quantification of BM B cells (CD19^+^ B220^+^) following E/R induction. **C** Frequencies of B cell progenitors upon E/R induction (pro B: CD19^+^ B220^low^ CD93^+^ CD43^+^ IgM^−^ IgD^−^; pre B: CD19^+^ B220^low^ CD93^+^ CD43^−^ IgM^−^ IgD^−^; Immature B: CD19^+^ B220^low/+^ CD93^+^ CD43^−^ IgM^+^ IgD^−^). **D** Quantification of phenotypic HSC and the SLAM DP compartments upon E/R induction. *n* = 5 for the control group and 7 for the E/R group. Student’s *t* test was used. Gene set enrichment analysis (GSEA) plots highlighting enrichment of an HSC signature (**E**) and depletion of cell cycle activity genes (**F**) in E/R-induced HSCs. **G** PB chimerism and multilineage contribution in mice transplanted with E/R-induced HSCs (or control HSCs), designated by CD45.2 expression. *n* = 7 mice/group. The transplanted animals were sacrificed after 18 weeks, their BM cells pooled, and unfractionated BM cells were re-transplanted into lethally irradiated WT recipients. PB chimerism and multilineage contribution are depicted in **H**. *n* = 5 mice per group. Student’s *t* test was used. **I** Automated WBC counts (left), and PB chimerism and lineage contribution (right) in the non-competitive transplantation experiment 12 weeks after Dox withdrawal. **J** PB chimerism and lineage distribution after 12 weeks of E/R induction (left) followed by 8 weeks of E/R removal (right). *n* = 5 mice per group. Ordinary one-way ANOVA test was used. Error bars denote mean ± SEM.

Since the acquisition of E/R is the initial event in the process of leukemogenesis, we induced iE/R mice for two weeks and characterized this “preleukemic” state in the BM (Fig. 1A). We observed a substantial decrease in CD19 ^+^ B cells and reductions at various B-cell developmental stages (Fig. 1B, C and Supplementary Fig. S1I). This was coupled with a marked increase in the frequency of phenotypic HSCs and the prominent emergence of a SLAM DP population (Fig. 1D and Supplementary Fig. S1J, K). Transcriptional profiling of the candidate E/R expressing HSCs established enrichments for genes linked to functional HSC activity (Fig. 1E and Supplementary Fig. S1L, M), with a parallel reduction of cell cycle-associated genes (Fig. 1F) [8]. Taken together, this initial characterization of the E/R preleukemic state provided functional and molecular evidence for compromised B-cell differentiation and a numerical expansion of phenotypic/candidate HSCs.

Previous studies have consistently observed a correlation between E/R expression and the proliferation of phenotypic HSCs [1, 3–5]. However, continuous expression of E/R in prior models confines work aimed at defining HSC activity. This is because E/R might restrict the multilineage differentiation capacity of HSCs, and/or might induce aberrant cellular phenotypes. Using our iE/R model, which enables rapid removal of E/R expression, we assessed the functional properties of the E/R-exposed HSCs. To assess this, we competitively transplanted 100 HSCs from uninduced (Control) or transiently induced (E/R) CD45.2 iE/R mice into CD45.1 WT irradiated hosts (Supplementary Fig. S1N). Long-term multilineage peripheral blood (PB) reconstitution was sustained for 16 weeks at similar levels between the two groups (Fig. 1G and Supplementary Fig. S1O, P) and was further maintained upon secondary transplantation (Fig. 1H). This demonstrates that transient E/R expression does not irreversibly impair normal HSC functionality.

To further assess the autonomous impact of E/R on hematopoiesis, we transplanted three million iE/R unfractionated BM cells into lethally irradiated WT recipients receiving either normal (WT) or Dox (E/R) food (Supplementary Fig. S1Q). In this non-competitive design, E/R expression markedly reduced PB white blood cell (WBC) counts and compromised reconstitution, particularly impacting lymphopoiesis (Fig. 1I). BM analysis revealed early differentiation blocks, with pronounced reductions of early MPP Ly progenitor cells and an almost complete absence of B cell progenitors (Supplementary Fig. S1R, S). Intriguingly, removal of E/R expression after 12 weeks restored donor-derived chimerism and normal B-cell differentiation (Fig. 1I and Supplementary Fig. S1R, S). These data established that E/R expression compromises hematopoietic reconstitution and lymphoid differentiation in vivo while allowing for HSCs to persist in the BM with retained function.

Intrigued by the observation that E/R HSCs could persist in vivo with preserved function, we assessed how E/R might affect the HSC competitiveness. For this, we transplanted an equal number of iE/R and WT unfractionated BM cells into lethally irradiated WT recipient mice. Upon analysis 12 weeks after transplantation, we could barely detect iE/R cells in the PB of the Dox-induced animals (Fig. 1J, left). Additionally, removing E/R failed to rescue the impaired reconstitution capacity of these cells (Fig. 1J, right). Therefore, despite the potential of E/R-expressing HSCs to persist long-term in the BM (Fig. 1I), they are ultimately outcompeted by WT HSCs.

**Fig. 2:**
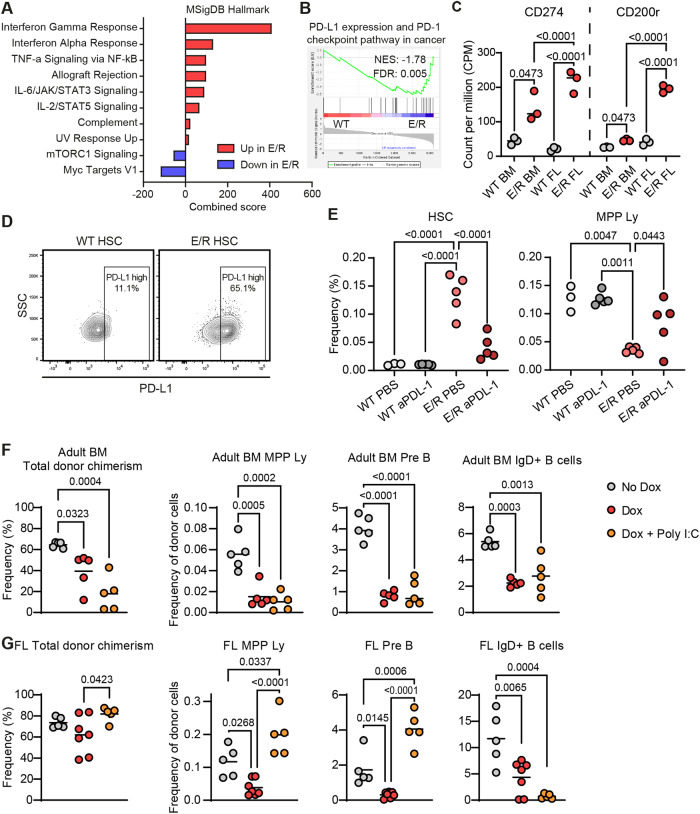
E/R fetal cells outperform E/R adult cells and gain a competitive advantage in response to the viral mimic poly I:C, allowing for the expansion of early HSPC and B cell compartments. **A** Up- or downregulated MSigDB Hallmark pathways from the common E/R dysregulated genes. Pathways with FDR values < 0.05 are displayed. *n* = 3 replicates/group. **B** GSEA plot of E/R HSCs (fetal and adult combined) versus their respective WT controls for PD-L1 pathway activation in cancer. **C** Log2 FC of CD274 (PD-L1) and CD200 receptor genes in E/R BM HSCs or E/R FL HSCs in comparison to their respective WT controls (left) and in E/R FL HSCs in comparison to E/R BM HSCs (right). **D** Representative FACS plots depicting the E/R-mediated increase in PD-L1 surface expression. *n* = 4 (for WT) and 7 (for E/R) mice, respectively. **E** Quantification of HSC and MPP Ly in WT and E/R mice after anti-PD-L1 therapy. *n* = 3–5 mice/group from two independent experiments. *p*-values < 0.05 are displayed (ordinary one-way ANOVA test). **F**, **G** Quantification of total donor chimerism and different BM cellular compartments in animals transplanted with E/R adult BM (**F**) or E/R E14.5 FL cells (**G**) with or without poly I:C. *n* = 7 mice for the E/R FL Dox and 5 mice for other groups. Mean and individual mice are shown. *p* values < 0.05 are displayed (ordinary one-way ANOVA).

E/R fusions arise in utero, forming preleukemic clones that can persist in the BM until adolescence [1]. However, in line with the poor competitiveness of iE/R HSCs (Fig. 1J), the prevalence of E/R leukemia drops dramatically in adulthood, suggesting that E/R preleukemic clones are largely outcompeted at this developmental stage. To explore the molecular program contributing to the prolonged persistence of fetal E/R cells compared to their adult counterparts, we isolated E14.5 fetal liver (FL) or adult BM HSCs from both WT and iE/R mice. These HSCs were cultured for four days under conditions that promote HSC activity, after which we performed RNA-sequencing analysis (RNA-seq) [9] (Supplementary Fig. S2A). By comparing E/R FL to WT FL and E/R BM to WT BM, along with E/R induction in vivo (related to Fig. 1), we identified genes differentially regulated by E/R (Supplementary Fig. S2B, C). E/R increased the expression of 73 genes across all three evaluated E/R conditions (Supplementary Fig. S2C), with approximately one-third having been previously identified as upregulated following RUNX1 knockout [10] (Supplementary Fig. S2D). This endorses that E/R corrupts differentiation at least in part by affecting normal RUNX1 targets [11, 12]. Consistent with previous studies [11–14], E/R-dysregulated genes associated with a significant depletion of MYC and mTORC1 signaling and enrichment of inflammatory pathways and major histocompatibility complex (MHC) class I antigen presentation (Fig. 2A and Supplementary Fig. S2E). This enrichment extended to the PD-L1 expression and PD-1 checkpoint pathway in cancer (Fig. 2B), as well as PD-L1 (CD274) and CD200 receptor expression in both E/R BM and E/R FL (Fig. 2C). The much higher enrichment of these genes in E/R fetal cells suggests a greater potential for immune evasion compared to E/R adult cells (Fig. 2C and Supplementary Fig. S2F).

To assess the functional implications of the PD-1/PD-L1 induction in response to E/R (Fig. 2D), we subjected mice to anti-PD-L1 treatment and monitored changes in BM hematopoietic progenitor cells (Supplementary Fig. S2G). While anti-PD-L1 treatment did not visibly affect the pool of early HSPCs in WT mice, it reduced the frequency of phenotypic HSCs and increased the frequencies of MPP Ly cells in the E/R setting (Fig. 2E), with milder effects on other compartments (Supplementary Fig. S2H, I).

Related to this, and in line with epidemiological evidence supporting infections as E/R transformation triggers, we finally tested whether E/R preleukemic cells might be favored in a setting of viral mimicry. We competitively transplanted three million iE/R BM or FL cells into irradiated hosts and administered polyinosinic:polycitidylic acid (poly I:C) intraperitoneally (IP) once a week for a month, followed by BM analysis one week after the final injection (Supplementary Fig. S2J). As expected, iE/R BM cells presented with reduced reconstitution capacity, with reductions in MPP Ly and all early B-cell stages, and these changes persisted after poly I:C treatment (Fig. 2F and Supplementary Fig. S2K). In contrast, the reconstitution ability of FL cells was less affected by E/R (Fig. 2G). Although FL cells are generally more efficient in lymphopoiesis compared to adult cells [15], E/R induction in FL cells still reduced MPP Ly and all investigated B-cell compartments (Fig. 2G and Supplementary Fig. S2L). Intriguingly, these phenotypes were substantially altered following poly I:C administration to FL E/R cells (Fig. 2G and Supplementary Fig. S2L). Notably, poly I:C treatment led to a remarkable increase in the pro-B and pre-B compartments of fetal E/R cells, surpassing WT levels by ~5- and 2.5-fold, respectively, albeit with less effects on later B cell differentiation stages (Fig. 2G and Supplementary Fig. S2L). Collectively, these data indicate that E/R fetal cells outperform their adult counterparts, particularly in response to poly I:C exposure. This lends support to an ontogeny-linked capacity of preleukemic E/R-expressing cells to differentiate into early B-cell progenitors. We speculate that these cells might subsequently be amenable to leukemic transformation.

In conclusion, our results shed light on two significant aspects: the persistence of E/R preleukemic clones and the reduced incidence of E/R leukemia in adulthood. By unraveling these phenomena, our study contributes to understanding the underlying mechanisms governing E/R leukemia dynamics and suggests immune modulation and checkpoint inhibitors as potential therapeutic approaches in E/R leukemia.

### Supplementary information

SUPPLEMENTAL MATERIAL

## Data Availability

The data generated in this study are publicly available at Gene Expression Omnibus GSE239344.
