# Supplementary material for: Ontogeny shapes the ability of ETV6::RUNX1 to enhance hematopoietic stem cell self-renewal and disrupt early lymphopoiesis
Source: Leukemia. 2024 Jan 19;38(2):455–9. doi: 10.1038/s41375-024-02149-2 (PMC10844086; doi:10.1038/s41375-024-02149-2)
Supplement: Supplementary file 1 — SUPPLEMENTAL MATERIAL [file 41375_2024_2149_MOESM1_ESM.pdf]

SUPPLEMENTAL INFORMATION

Materials and methods

Generation of iE/R mice

A human E/R fusion gene, coupled to an AviTag, was subcloned into the EcoRI site of the pBS31 vector (a kind gift from Rudolph Jaenisch) and used to target the 3' UTR of the Col1a1 locus under a Tetracycline-regulated promoter, along with an M2 reverse Tetracycline transactivator (M2-rtTA) in the Rosa26 locus of KH2 embryonic stem cells (ESCs) [1]. Following karyotyping, determination of transgene integration into the Col1a1 locus, and verification of its inducibility, ESCs were injected into morulas to generate chimeric mice (Lund University Transgenic Animal Facility). One high-level chimera was bred to WT C57bl/6 mice and the colony was subsequently maintained by breeding mice homozygous for both the E/R fusion gene and the Rosa26-rTTA. E/R was induced in vivo by administering doxycycline (Dox) food (2g/kg; ssniff Spezialdiäten). Mice were maintained in the animal facilities at the Biomedical Center of Lund University, and all animal experiments were performed with the approval of the local ethics committee.

Immunophenotypic analysis, cell sorting, and HSC transplantation

Identification and isolation of different cell populations were performed as previously described [2,3] using the following antibodies.

ANTIBODY	SOURCE	IDENTIFIER
CD117 (c-Kit)-APC	Biolegend	Cat# 105812, RRID: AB_1282964
CD117-APC/eFluor 780	eBioscience	Cat# 47-1171-82, RRID: AB_1272177

CD4-Biotin	Sony	Cat# 1102020, RRID: AB_1848918
CD8-Biotin	Sony	Cat# 1103520, RRID: AB_962670
Ly-6G/Ly-6C (Gr-1)-Biotin	BioLegend	Cat# 108404, RRID: AB_313369
Ly6G/Ly6C (Gr-1)-PE/Cy5	BioLegend	Cat# 108410, RRID: AB_313375
TER-119-Biotin	BioLegend	Cat# 116203, RRID: AB_313704
CD3-Alexa Fluor 700	Sony	Cat# 1101080, RRID: N/A
CD3-PE/Cy5	Sony	Cat# 1101550, RRID: N/A
Streptavidin - Brilliant Violet 605	Sony	Cat# 2626145
Ly-6A/E (Sca-1)-Pacific Blue	BioLegend	Cat# 122520, RRID: AB_2143237
CD48-FITC	Sony	Cat# 1117020, RRID: N/A
CD48-Alexa Fluor 700	Sony	Cat# 1117130, RRID: N/A
CD150-PE/Cy7	Sony	Cat# 1179570, RRID: N/A
CD150-PE	BioLegend	Cat# 115904, RRID: AB_313683
CD201-PE	Sony	Cat# 1307520, RRID: N/A
CD201-APC	eBioscience	Cat# 17-2012-82, RRID: AB_10717805
CD11b-APC	Sony	Cat# 1106060, RRID: N/A
CD127-Biotin	Sony	Cat# 1275030, RRID: N/A
CD135-PE	Sony	Cat# 1276530, RRID: N/A
CD19-PE/Cy7	Sony	Cat# 1177600, RRID: N/A
CD25-FITC	BD Bioscience	Cat# 553072, RRID: AB_394604
CD43-Brilliant Violet 605	BD Bioscience	Cat# 747726, RRID: AB_2872201
CD45.1-Alexa Fluor 700	Sony	Cat# 1153620, RRID: N/A
CD45.1-Brilliant Violet 650	Sony	Cat# 1153680, RRID: N/A

CD45.2-Brilliant Violet 785	Sony	Cat# 1149195, RRID: N/A
CD45.2-FITC	Sony	Cat# 1149030, RRID: N/A
CD45R/B220-Alexa Fluor 700	Sony	Cat# 1116160, RRID: N/A
B220-Biotin	BioLegend	Cat# 103203, RRID: AB_312988
B220-PE/Cy5	Sony	Cat# 1116050, RRID: N/A
CD93-Brilliant Violet 785	BD Bioscience	Cat# 740941, RRID: AB_2740571
IgD-APC	Sony	Cat# 2628570, RRID: N/A
IgM-Brilliant Violet 421	Sony	Cat# 2632590, RRID: N/A

For primary HSC transplantations, LSK SLAM cells were isolated by flow cytometry from CD45.2+ iE/R mice and competitively transplanted into lethally irradiated (900 rad) ~8 weeks old CD45.1 females 3–4 hr after irradiation. Peripheral blood (PB) analyses were conducted as described previously [4]. Cells were sorted on a FACS Aria II/III (Becton Dickinson).

Competitive and non-competitive BM transplantations and serial transplantations

For non-competitive transplantations, three million iE/R unfractionated bone marrow (BM) cells were transplanted into lethally irradiated ~8 weeks old CD45.1 C57bl/6 females. For the competitive transplantation experiments, one million iE/R unfractionated BM cells were transplanted together with one million CD45.1 unfractionated BM cells into lethally irradiated CD45.1 C57bl/6 mice. To induce E/R expression, recipients were provided Dox-containing diet 5 days before transplantation and onwards. Serial transplantations were performed by pooling unfractionated BM cells from the primary transplanted animals. Three million cells were then transplanted into a set of new lethally irradiated CD45.1 C57bl/6 mice.

iE/R *Pax5*$^{+/-}$ *Ebf1*$^{+/-}$ mice generation and leukemia development

Homozygous iE/R mice were crossed with *Pax5*$^{+/-}$ *Ebf1*$^{+/-}$ mice [5]. Following genotyping to obtain iE/R *Pax5*$^{+/-}$ *Ebf1*$^{+/-}$ mice, two million unfractionated BM cells from the generated strains were transplanted into lethally irradiated recipients. Mice were monitored for signs of leukemia development according to the Bethesda classification for murine lymphoid malignancies [6]. Diseased mice were subjected to necroscopic and FACS analyses. Survival rates were plotted using the Kaplan-Meier method.

RNA seq on in vivo E/R HSC

HSCs (1,500 cells/replicate) were sorted from control or E/R-induced mice, n = 3 replicates/group. RNA extraction was performed using the Norgen single-cell RNA purification kit (51800, Norgen Biotech). Library preparation was performed using SMARTer Stranded Total RNA-Seq Kit v2 – Pico Input Mammalian (634418, TaKaRa). Library concentration was measured using QuantIT 1X dsDNA HS Assay Kit (Q33232, Thermo Fisher), and quality was assessed on 5200 Fragment Analyzer™ 12-capillary (M3510AA, Agilent) using DNF-930 dsDNA Reagent Kit, 75 bp – 20 000 bp (K0500, Agilent). Sequencing was performed using NovaSeq 6000 S2 Reagent Kit (2x100 paired-end cycles) (20012861, Illumina). Demultiplexing was performed using bcl2fastq2 and alignment to the Mouse GRCm38 was performed using the HISAT2 software. StringTie was used for assembly and quantification. Differential gene expression analysis was performed using DESeq2. GSEA was performed as previously described [4,7].

RNA seq on in vitro-induced WT or E/R BM and FL HSC

Fetal liver (FL) (E14.5) and BM (8 weeks) HSCs were sorted from WT or iE/R and cultured for three days in ex vivo HSC expansion conditions [8] with 1 µg/mL Dox (Sigma-Aldrich). RNA

extraction and sequencing were performed as described above. Reads were aligned to the Ensembl release 76 primary assembly with STAR version 2.5.1a. Isoform expression of known Ensembl transcripts was estimated with Salmon version 0.8.2. The ribosomal fraction, known junction saturation, and read distribution over known gene models were quantified with RSeQC version 2.6.2.

Gene counts were imported into the R/Bioconductor package EdgeR and TMM normalization size factors were calculated. Counts were imported into the R/Bioconductor package Limma. Ribosomal genes and genes not expressed in the smallest group size minus one sample greater than one count-per-million were excluded from further analysis. Weighted likelihoods based on the observed mean-variance relationship were calculated for all samples with the voomWithQualityWeights. Residual standard deviation plots of every gene to their average log-count were assessed. Differential expression analysis was performed, and results were filtered for genes with Benjamini-Hochberg false-discovery rate adjusted p-values ≤0.05. Gene ontology analysis was performed using DAVID Bioinformatics Resources v6.8.

In vivo anti-PD-L1 treatment

WT and iE/R mice were i.p. injected with anti-PD-L1 antibody (10 mg/kg, clone 10F.9G2, BioXCell, RRID: AB_2927503, diluted in PBS) or PBS at days 0, +4, +7, +11 and +14. BM analysis was performed 4 days after the last injection. iE/R mice were provided Dox food from day 0 throughout the experiment.

Poly I:C injections

Three million iE/R unfractionated BM (8 weeks old) or FL (E14.5) cells were transplanted with 300,000 competitor WT cells into lethally irradiated WT mice. Apart from the No Dox group, recipients were provided Dox food to induce E/R expression. One month later, E/R-induced

mice received weekly IP injections of polyinosinic:polycitidylic acid (poly I:C) (10 µg/g), or PBS as a control, for 4 consecutive weeks. BM analysis was performed one week after the last injection.

Quantitative RT-PCR

Quantitative reverse-transcription PCR (qRT-PCR) was performed as described previously [4] using the listed primers and signals were normalized to β-actin mRNA expression levels.

β Actin Fw	CCACAGCTGAGAGGCAAATC	Integrated DNA Technologies
β Actin Rev	CTTCTCCAGGGAGGAAGAGG	Integrated DNA Technologies
ETV6::RUNX1 Fw	CCTCTCTCATCGGGAAGACC	Integrated DNA Technologies
ETV6::RUNX1 Rev	CAACGCCTCGCTCATCTTG	Integrated DNA Technologies

Statistical analysis

Data analysis was performed using GraphPad Prism (RRID: SCR_002798) and Microsoft Excel. FACS data were analyzed with FlowJo (RRID: SCR_008520). Significance was calculated by Student's two-tailed t-test, ordinary one-way ANOVA, or the log-rank test (Mantel-Cox test) for Kaplan-Meier curves. Statistical details of experiments can be found in figure legends. ∗$p < 0.05$, ∗∗$p < 0.01$, ∗∗∗$p < 0.001$; n.s., not significant.

Supplementary references

1 Beard C, Hochedlinger K, Plath K, Wutz A, Jaenisch R. Efficient method to generate single-copy transgenic mice by site-specific integration in embryonic stem cells. *genesis* 2006; 44: 23–28.

2 Challen GA, Pietras EM, Wallscheid NC, Signer RAJ. Simplified murine multipotent progenitor isolation scheme: Establishing a consensus approach for multipotent progenitor identification. *Experimental Hematology* 2021; 104: 55–63.

3 Norddahl GL, Pronk CJ, Wahlestedt M, Sten G, Nygren JM, Ugale A *et al.* Accumulating mitochondrial DNA mutations drive premature hematopoietic aging phenotypes distinct from physiological stem cell aging. *Cell Stem Cell* 2011; 8: 499–510.

4 Eldeeb M, Yuan O, Guzzi N, Thi Ngoc PC, Konturek-Ciesla A, Kristiansen TA *et al.* A fetal tumor suppressor axis abrogates MLL-fusion-driven acute myeloid leukemia. *Cell Reports* 2023; 42: 112099.

5 Prasad MAJ, Ungerbäck J, Åhsberg J, Somasundaram R, Strid T, Larsson M *et al.* Ebf1 heterozygosity results in increased DNA damage in pro-B cells and their synergistic transformation by Pax5 haploinsufficiency. *Blood* 2015; 125: 4052–4059.

6 Morse HC. Bethesda proposals for classification of lymphoid neoplasms in mice. *Blood* 2002; 100: 246–258.

7 Subramanian A, Tamayo P, Mootha VK, Mukherjee S, Ebert BL, Gillette MA *et al.* Gene set enrichment analysis: A knowledge-based approach for interpreting genome-wide expression profiles. *Proceedings of the National Academy of Sciences* 2005; 102: 15545–15550.

8 Wilkinson AC, Ishida R, Kikuchi M, Sudo K, Morita M, Crisostomo RV *et al.* Long-term ex vivo haematopoietic-stem-cell expansion allows nonconditioned transplantation. *Nature* 2019; 571: 117–121.

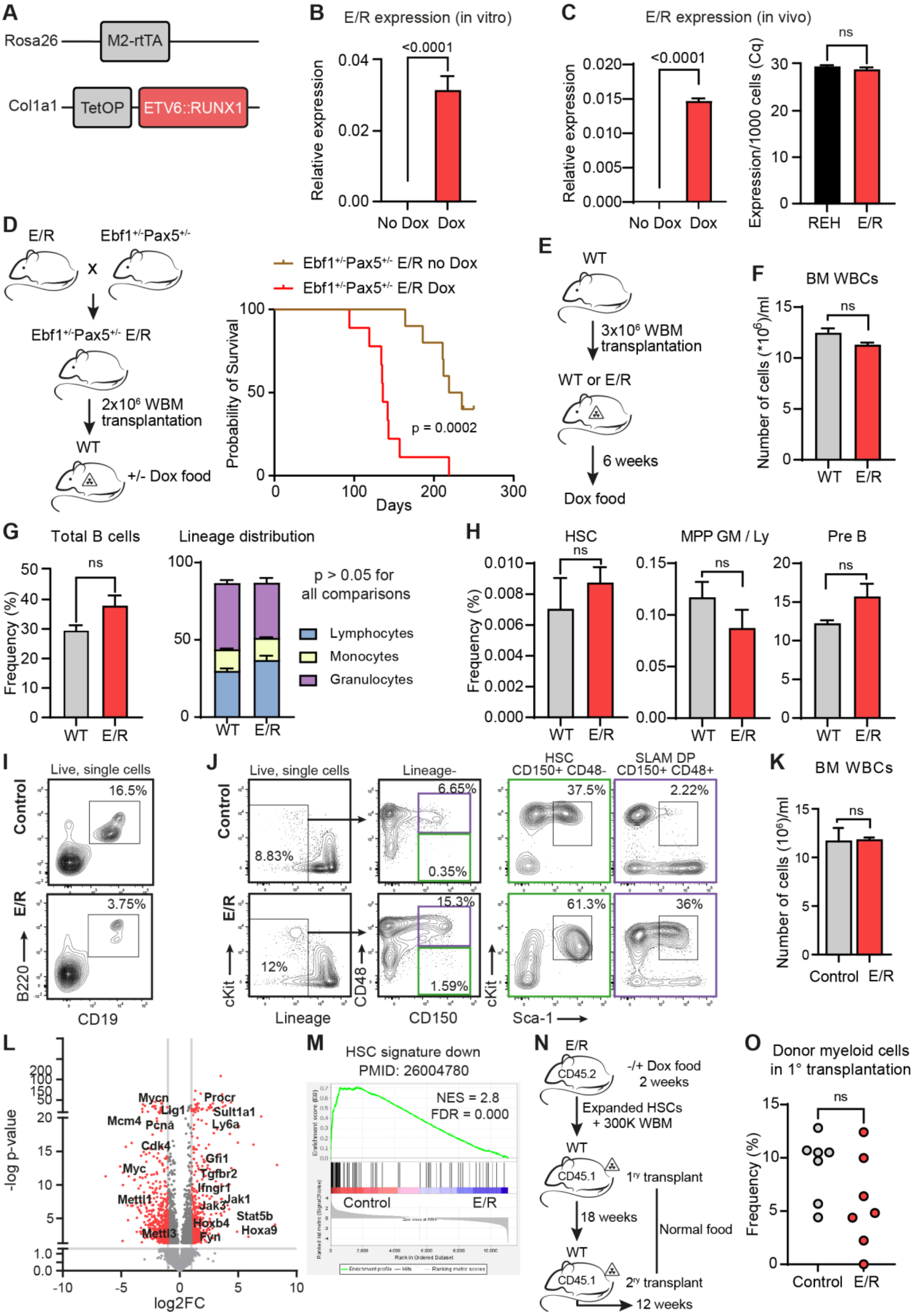
A
Rosa26
M2-rtTA
Col1a1
TetOP
ETV6::RUNX1
B
E/R expression (in vitro)
Relative expression
<0.0001
No Dox
Dox
C
E/R expression (in vivo)
Relative expression
<0.0001
Expression/1000 cells (Cq)
ns
REH
E/R
D
E/R
Ebf1+/-Pax5+/-
Ebf1+/-Pax5+/- E/R
2x10⁶ WBM transplantation
WT
+/- Dox food
Ebf1+/-Pax5+/- E/R no Dox
Ebf1+/-Pax5+/- E/R Dox
Probability of Survival
p = 0.0002
Days
E
WT
3x10⁶ WBM transplantation
WT or E/R
6 weeks
Dox food
F
BM WBCs
Number of cells (*10⁶)/ml
G
Total B cells
Lineage distribution
Frequency (%)
p > 0.05 for all comparisons
Lymphocytes
Monocytes
Granulocytes
H
HSC
MPP GM / Ly
Pre B
I
Live, single cells
Control
E/R
B220
CD19
J
Lineage-
HSC CD150+ CD48-
SLAM DP CD150+ CD48+
cKit
Lineage
CD48
CD150
Sca-1
K
BM WBCs
Control
L
-log p-value
log2FC
Mycn
Procr
Lig1
Sult1a1
Mcm4
Pcna
Ly6a
Cdk4
Gfi1
Myc
Tgfbr2
Ifngr1
Mettl1
Jak1
Jak3
Stat5b
Hoxb4
Hoxa9
Mettl3
Fyn
M
HSC signature down PMID: 26004780
NES = 2.8
FDR = 0.000
N
-/+ Dox food 2 weeks
CD45.2
Expanded HSCs + 300K WBM
CD45.1
1ry transplant
18 weeks
Normal food
2ry transplant
12 weeks
O
Donor myeloid cells in 1° transplantation

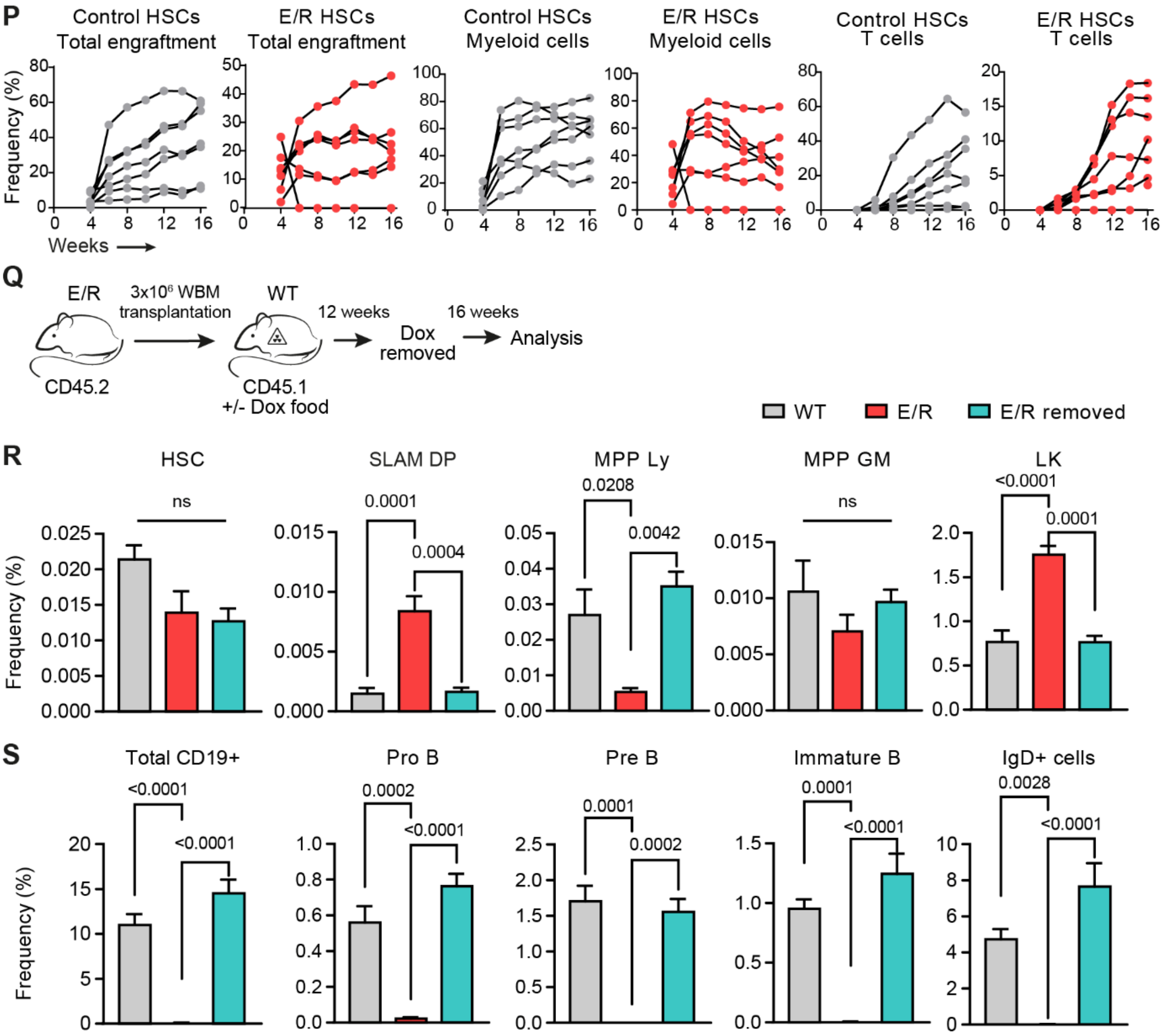

Figure S1. Generation and initial validation of a Doxycycline-inducible mouse model for E/R-ALL. A) Schematic depiction of the inducible E/R mouse model (iE/R). B) qRT-PCR was performed on RNA from iE/R Kit-enriched BM cells after three days of culture, confirming the selective expression of E/R upon Dox administration. Expression levels were normalized to Beta-actin. n = 4 - 6 replicates/condition. C) (Left) qRT-PCR was performed on RNA from iE/R BM cells isolated from uninduced or two-weeks-induced mice, confirming the selective expression of E/R upon Dox administration. Expression levels were normalized to Beta-actin. n = 3 replicates/condition. (Right) Expression levels of E/R in 1000 cells from iE/R-induced mice or REH human cell line. n = 3 replicates/group. D) Experimental outline to determine the impact of E/R on *Ebf1*$^{-/+}$ *Pax5* $^{-/+}$ driven B-ALL (left), and Kaplan-Meier survival curve of the

transplanted mice with or without E/R induction (right). n = 10 mice/group. A Log-rank (Mantel-Cox) test was used to determine significance. E) Experimental outline to assess the impact of E/R expression in non-hematological cells. n = 5 mice/group. F) WBC counts of BM samples. G) Frequency of donor-derived cells in B cell lineage (left) and BM lineage distribution of donor cells (right). H) Frequency of donor-derived HSC, MPP GM/Ly, and pre-B cells in the BM. I) Representative FACS plots (left) of BM B cells ($CD19^+$ $B220^+$) following induction of iE/R mice (lower panel) and control (upper panel). J) Representative FACS plots showing the impact of E/R expression on the frequency of phenotypic HSCs and the SLAM DP population in the BM of control (upper panels) and E/R induced (lower panels) mice. K) WBC counts of BM samples following iE/R mice induction. Error bars denote mean ± SEM. Student's t-test was used in C-F, and p-values < 0.05 are displayed. L) Volcano plot highlighting differentially expressed mRNA targets (red) upon E/R induction. Grey lines represent log2FC of −1 and +1. n = 3 replicates/group. M) GSEA plot highlighting enrichment of a non-HSC associated signature in control compared to E/R-induced HSCs. N) Experimental strategy for HSC transplantation. O) Frequency of donor-derived myeloid cells in the PB of primary transplantations. P) PB chimerism and multilineage contribution of individual mice transplanted with E/R-induced HSCs (or control HSCs), designated by CD45.2 expression. Q) Experimental strategy to assess reversibility of E/R. R) Frequency of HSPC subsets in the BM 16 weeks following Dox withdrawal. n = 5 mice in the no Dox group and 4 in each of the other groups. S) Frequency of different BM B cell progenitor subsets. n = 4 mice/group. p-values < 0.05 are displayed (ordinary one-way ANOVA). Error bars denote mean ± SEM.

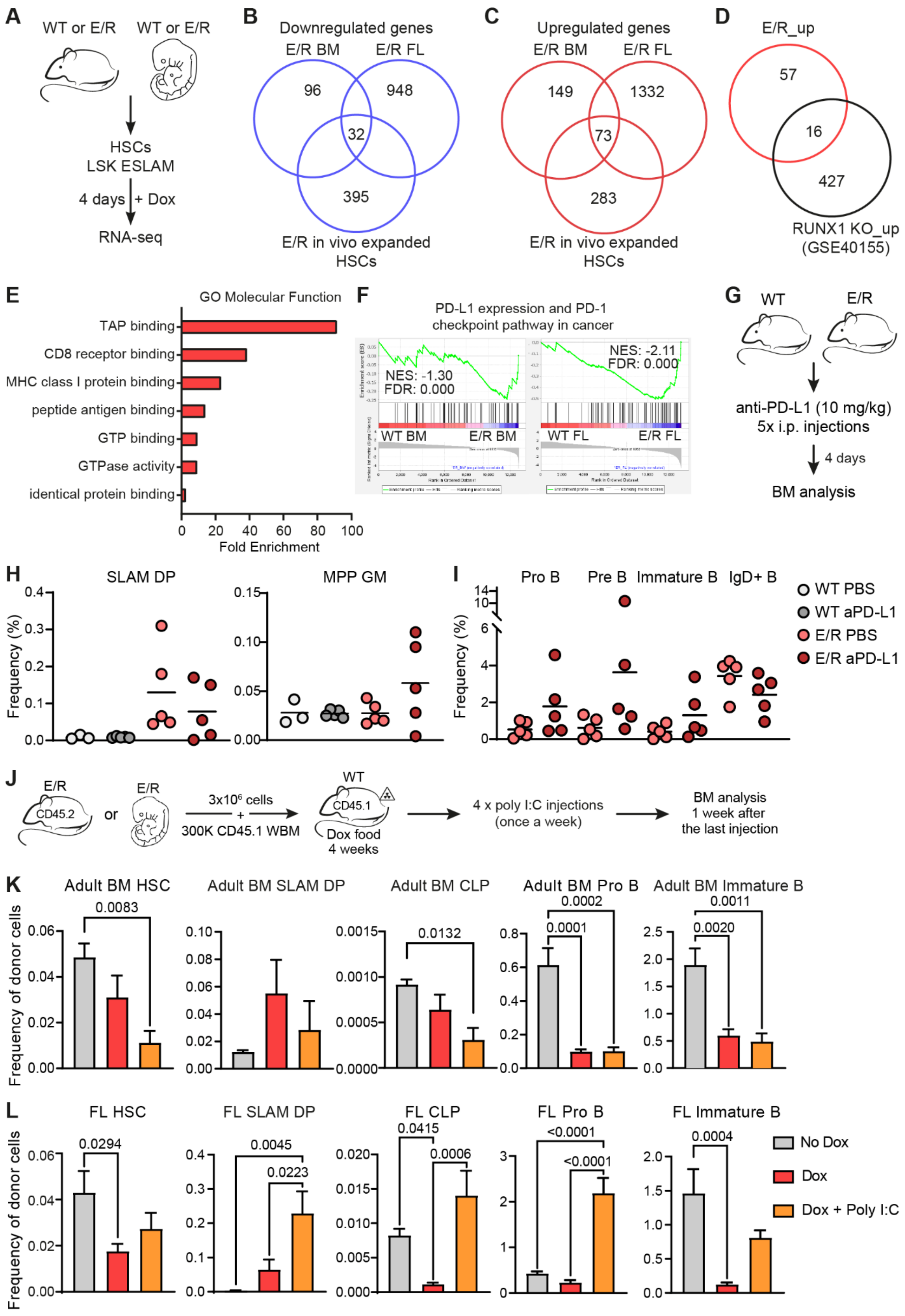
A
WT or E/R
WT or E/R
HSCs
LSK ESLAM
4 days + Dox
RNA-seq
B
Downregulated genes
E/R BM
E/R FL
96
948
32
395
E/R in vivo expanded HSCs
C
Upregulated genes
E/R BM
E/R FL
149
1332
73
283
E/R in vivo expanded HSCs
D
E/R_up
57
16
427
RUNX1 KO_up (GSE40155)
E
GO Molecular Function
TAP binding
CD8 receptor binding
MHC class I protein binding
peptide antigen binding
GTP binding
GTPase activity
identical protein binding
Fold Enrichment
F
PD-L1 expression and PD-1 checkpoint pathway in cancer
NES: -1.30
FDR: 0.000
NES: -2.11
FDR: 0.000
WT BM
E/R BM
WT FL
E/R FL
G
WT
E/R
anti-PD-L1 (10 mg/kg)
5x i.p. injections
4 days
BM analysis
H
SLAM DP
MPP GM
Frequency (%)
I
Pro B
Pre B
Immature B
IgD+ B
WT PBS
WT aPD-L1
E/R PBS
E/R aPD-L1
J
E/R
CD45.2
or
E/R
3x10^6 cells
+
300K CD45.1 WBM
WT
CD45.1
Dox food
4 weeks
4 x poly I:C injections
(once a week)
BM analysis
1 week after
the last injection
K
Adult BM HSC
Adult BM SLAM DP
Adult BM CLP
Adult BM Pro B
Adult BM Immature B
Frequency of donor cells
0.0083
0.0132
0.0002
0.0001
0.0011
0.0020
L
FL HSC
FL SLAM DP
FL CLP
FL Pro B
FL Immature B
Frequency of donor cells
0.0294
0.0045
0.0223
0.0415
0.0006
<0.0001
<0.0001
0.0004
No Dox
Dox
Dox + Poly I:C

Figure S2. E/R expression heightens PD-L1 activity and increases the competitive advantage of fetal cells in response to the viral mimic poly I:C. A) Experimental outline. n = 3 replicates/group. B) and C) Overlap between differentially downregulated (B) or upregulated (C) genes in E/R BM, E/R FL, and E/R expanded HSCs (related to Fig. 1), in comparison to their respective WT controls (log2FC +/-0.85-1). D) Overlap between common E/R upregulated genes and genes upregulated in LSK cells following knockout of RUNX1 (GSE40155). E) Molecular function prediction for the common upregulated genes upon E/R induction. F) GSEA plot of E/R BM HSCs (left) or E/R FL HSCs (right) versus their respective WT controls for PD-L1 pathway activation in cancer. G) Experimental setup to assess the effects of in vivo PD-L1 blockade on E/R preleukemic cells. H) Quantification of SLAM DP and MPP GM in WT and E/R mice after anti-PD-L1 therapy. I) Quantification of B-cell differentiation stages in E/R mice following anti-PD-L1 therapy. J) Experimental strategy to assess the impact of poly I:C on E/R adult BM or E14.5 FL cells. K-L) Bar plots quantifying different HSPCs and B-cell progenitor compartments in the BM of animals transplanted with E/R adult BM (K) or E/R FL cells (L). n = 5 mice/group.
